# Decreased autophagy: a major factor for cardiomyocyte death induced by *β*_1_-adrenoceptor autoantibodies

**DOI:** 10.1038/cddis.2015.237

**Published:** 2015-08-27

**Authors:** L Wang, H Hao, J Wang, X Wang, S Zhang, Y Du, T Lv, L Zuo, Y Li, H Liu

**Affiliations:** 1 Department of Pathology, Shanxi Medical University, Taiyuan, Shanxi, PR China; 2 Department of Orthopaedics, Shanxi Dayi Hospital (Shanxi Academy of Medical Sciences), Taiyuan, Shanxi, PR China; 3 Department of Physiology, Shanxi Medical University, Taiyuan, Shanxi, PR China; 4 Department of Physiology and Pathophysiology, School of Basic Medical Sciences, Capital Medical University, Beijing, PR China

## Abstract

Cardiomyocyte death is one major factor in the development of heart dysfunction, thus, understanding its mechanism may help with the prevention and treatment of this disease. Previously, we reported that anti-*β*_1_-adrenergic receptor autoantibodies (*β*_1_-AABs) decreased myocardial autophagy, but the role of these in cardiac function and cardiomyocyte death is unclear. We report that rapamycin, an mTOR inhibitor, restored cardiac function in a passively *β*_1_-AAB-immunized rat model with decreased cardiac function and myocardial autophagic flux. Next, after upregulating or inhibiting autophagy with Beclin-1 overexpression/rapamycin or RNA interference (RNAi)-mediated expression of Beclin-1/3-methyladenine, *β*_1_-AAB-induced autophagy was an initial protective stress response before apoptosis. Then, decreased autophagy contributed to cardiomyocyte death followed by decreases in cardiac function. In conclusion, proper regulation of autophagy may be important for treating patients with *β*_1_-AAB-positive heart dysfunction.

Heart dysfunction is the terminal stage of various cardiovascular diseases, and it is characterized by a complicated etiology and high mortality. Recent studies indicate that cardiomyocyte death was a leading contributor to the development of heart dysfunction.^1^ Because systolic and diastolic function is directly affected by myocardial cell loss, understanding how cardiomyocyte death occurs will inform treatment strategies to prevent or treat heart dysfunction.

Since the 1990s, studies have revealed that diverse cardiovascular diseases are correlated to anti-*β*_1_-adrenergic receptor autoantibodies (*β*_1_-AABs).^2, 3^ We reported that *β*_1_-AABs were induced by myocardial remodeling in heart dysfunction,^4^ and that its long-term presence significantly decreased cardiac function *in vivo*.^5^ *β*_1_-AABs also caused cell death of cultured adult rat ventricular myocytes and this was attributed to apoptosis.^6^ Recently, work from our laboratory^7^ and others^8^ indicated that *β*_1_-AABs induced myocardial apoptosis. However, *β*_1_-AAB-induced cardiomyocyte death was not completely reversed with the caspase inhibitor Z-VAD-fmk,^6^ indicating that other factors were involved in *β*_1_-AAB-induced cardiomyocyte death.

Presently, we observed that *β*_1_-AABs decrease myocardial autophagy that maintains cellular homeostasis.^9^ Deficiencies in autophagy allow the accumulation of damaged, denatured or aging proteins^10^ and organelles,^11^ and this will cause cell death. To date, the role of *β*_1_-AAB-induced changes in autophagy as related to cardiac function and cardiomyocyte death is unclear. Therefore, we characterized *β*_1_-AAB-induced changes in myocardial autophagy and identified a role for this in cardiac function and cardiomyocyte death. Our data will inform future studies of *β*_1_-AAB-positive heart dysfunction and suggest a treatment window for autophagy regulation.

## Results

### *β*_1_-AABs caused the decrease of cardiac function in passively immunized rats

Rats were passively immunized by injecting *β*_1_-AABs (2 *μ*g/g), once every 10 days, for 80 days. Before each immunization, serum *β*_1_-AABs were measured and it increased 20 days after passive immunization. Serum *β*_1_-AABs remained stable until the end of the experiment in the *β*_1_-AAB group compared with the control group (Supplementary Figure S1a).

Meanwhile, cardiac function was measured 40 and 60 days after passive immunization, and there was no significant difference in left ventricular function between the immunized and control groups. However, animals had significantly decreased left ventricular systolic pressure (LVSP), maximal positive and negative values of the instantaneous first derivative of left ventricular pressure (+dP/dt_max_ and −dP/dt_max_) and significantly increased left ventricular end diastolic pressure (LVEDP) 80 days after passive immunization in the *β*_1_-AAB group compared with the control group (Supplementary Figure S1b–e), indicating that a long-term presence of *β*_1_-AABs could cause a significant decrease in cardiac function.

### Decreased autophagy had a role in decreased cardiac function induced by *β*_1_-AABs

**Figure 1:**
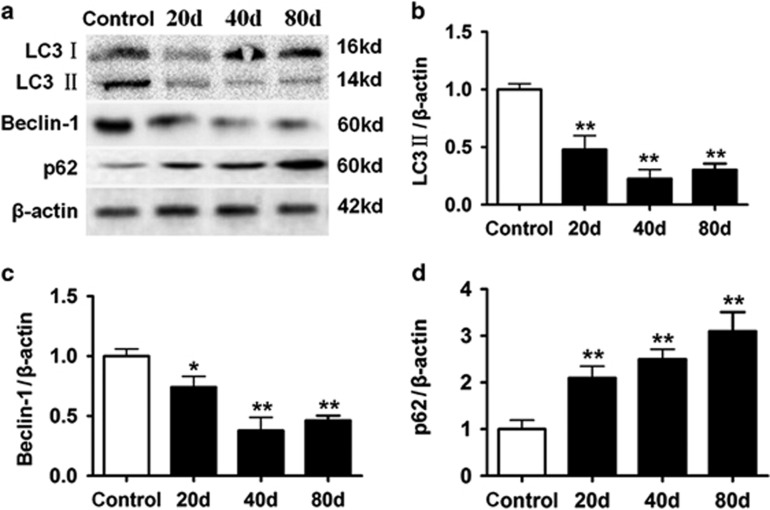
Change in autophagic flux induced by *β*_1_-AABs in myocardial tissues at 20, 40, and 80 days after passive immunization. (**a**) Representative western blots of autophagy markers LC3, Beclin-1, and p62 in lysates from control and *β*_1_-AAB rats. Each lane represents an individual animal. (**b**–**d**) Quantification of western blot data from (**a**). Data are presented as means±S.D. (*n*=6 per group). *P<0.05; **P<0.01

In this study, Beclin-1, an important gene in autophagic regulation, and LC3, a protein marker for autophagy, were selected to measure autophagy. Significantly decreased LC3 and Beclin-1 protein were observed 20 days after passive immunization and lower levels persisted 40 and 80 days after passive immunization in the *β*_1_-AAB group (Figures 1a–c), indicating that *β*_1_-AABs may lead to decreased autophagy in myocardial tissues. To confirm the results, p62 protein, which was degraded within the autolysosomes, was used as a marker of autophagic flux. At 20 days after immunization, p62 protein in cardiac tissue increased significantly in the *β*_1_-AAB group compared with control groups and p62 remained relatively high at 40 and 80 days (Figures 1a and d), indicating a defect in autophagic flux in the presence of *β*_1_-AABs.

**Figure 2:**
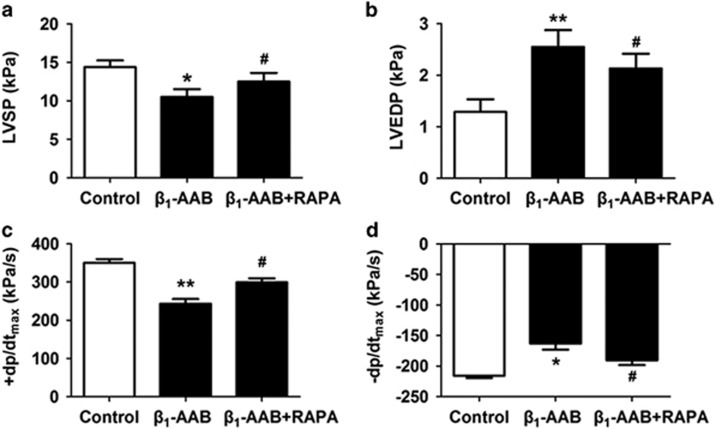
*β*_1_-AAB-induced decrease in cardiac function was reversed by upregulating autophagy using mTOR inhibitor rapamycin (RAPA). Cardiac function was measured with LVSP (**a**), LVEDP (**b**), +dP/dt_max_ (**c**), and −dP/dt_max_ (**d**). +dP/dt_max_ means maximal positive values of the instantaneous first derivative of left ventricular pressure and −dP/dt_max_ means negative values of the instantaneous first derivative of left ventricular pressure. LVSP and +dP/dt_max_ are the parameters of systolic function. LVEDP and −dP/dt_max_ are the indicators of diastolic function (*n*=6 per group). Data are expressed as means±S.D. *P<0.05, **P<0.01 *versus* Control; ^#^*P*<0.05 *versus* β_1_-AAB group

Rapamycin (RAPA), an mTOR inhibitor, is often used to upregulate autophagy.^12^ In this experiment, it was used to improve autophagy in rat myocardial tissue (Supplementary Figure S2). Significantly improved left ventricular function was observed in rats pretreated with rapamycin compared with those only passively immunized in the *β*_1_-AAB group (Figures 2a–d), suggesting that upregulating autophagy may reverse decreased cardiac function induced by *β*_1_-AABs and that insufficient autophagy caused by the long-term presence of *β*_1_-AABs may be associated with decreased cardiac function.

### *β*_1_-AABs caused the death of H9c2 cells

**Figure 3:**
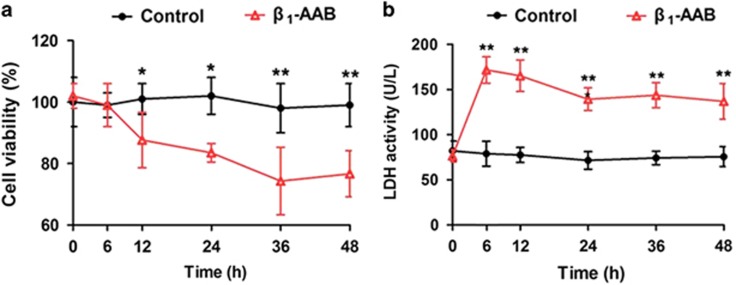
Effect of *β*_1_-AABs stimulation on H9c2 myocardial cells at different time points. H9c2 cells were treated with *β*_1_-AABs (1 *μ*mol/l) or negative IgGs (1 *μ*mol/l) at different time points. (**a**) Cell viability was measured by CCK-8 assay (*n*=16, means±S.D.) *P<0.05; **P<0.01. (**b**) LDH activity was measured to evaluate the extent of cell damage (*n*=16, means±S.D.) **P<0.01

Cell viability was measured to reflect the effects of *β*_1_-AABs stimulation of different durations. Significantly decreased cell viability was observed 12 h after *β*_1_-AABs stimulation and at a minimum of 36 h after *β*_1_-AABs stimulation (Figure 3a), indicating that *β*_1_-AABs may cause the death of myocardial cells.

LDH is released when cell membranes lyse, and it is an indicator of cell damage. Significantly increased LDH activity was observed at 6 h which lasted till the end of the experiment compared with the control group (Figure 3b), suggesting that *β*_1_-AABs may directly damage H9c2 cells.

### Decreased autophagy was critical for cardiomyocyte death

**Figure 4:**
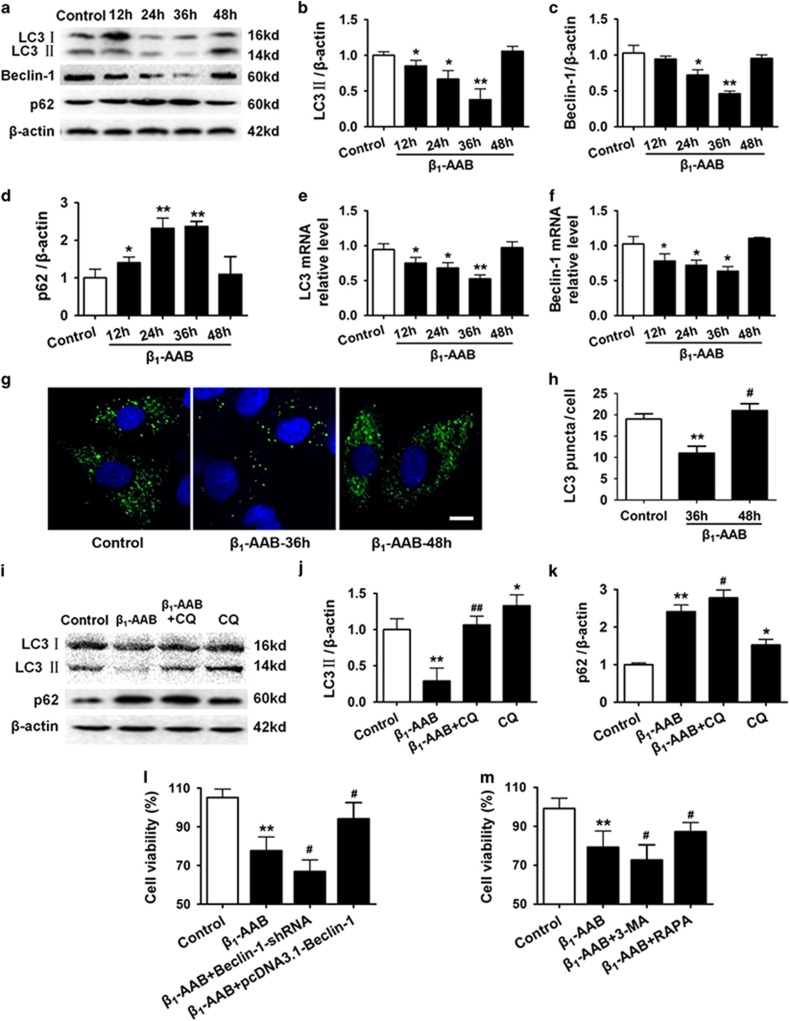
Change of autophagic flux induced by *β*_1_-AABs in H9c2 myocardial cells and its effect on cardiomyocyte death. (**a**) H9c2 myocardial cells were treated with *β*_1_-AABs or negative IgGs at 0, 12, 24, 36, and 48 h. Western blot indicated protein expression of LC3, Beclin-1, and p62 in H9c2 cells. (**b**–**d**) Quantification of western blot data from (**a**). Data are expressed as means±S.D. (*n*=6 per group). *P<0.05; **P<0.01. (**e** and **f**) Real-time PCR was used to measure LC3 and Beclin-1 mRNA expression in H9c2 cells. Data are expressed as means±S.D. (*n*=6 per group). *P<0.05; **P<0.01. (**g**) Representative images of immunofluorescence staining for LC3 (green) and DAPI (blue) in H9c2 cells. Scale bar was 10 *μ*m. (**h**) A statistical analysis by counting LC3 puncta in 20 different fields. The number of LC3 puncta/cell was evaluated as the total number of dots (green) divided by the number of nuclei (blue) in each microscopic field. Data are expressed as means±S.D. (*n*=6 per group). **P<0.01 *versus* Control; ^#^*P*<0.05 *versus β*_1_-AAB group. (**i**) H9c2 cells were pretreated with/or without 20 *μ*M chloroquine (CQ, an inhibitor of autophagy), and immunoblotting assays were performed with LC3B or p62 antibodies at 36 h after *β*_1_-AABs stimulation. (**j** and **k**) Quantification of western blot data from (**i**). Data are expressed as means±S.D. (*n*=6 per group). *P<0.05, **P<0.01 *versus* Control; ^#^*P*<0.05, ^##^*P*<0.01 *versus β*_1_-AAB group. (**l** and **m**) Change in cell viability 36 h after *β*_1_-AABs stimulation when myocardial autophagy was upregulated by overexpressing Beclin-1/RAPA or inhibited by RNA interference against Beclin-1/3-MA. Data are expressed as means±S.D. (*n*=8 per group). **P<0.01 *versus* Control; ^#^*P*<0.05 *versus β*_1_-AAB group

LC3 and Beclin-1 were used to indicate autophagy, and Beclin-1 and LC3 protein and mRNA in H9c2 myocardial cells were measured 0, 12, 24, 36, and 48 h after *β*_1_-AABs stimulation using western blot and real-time PCR. *In situ* expression of LC3 protein was measured with immunostaining. Beclin-1 protein decreased 24 h after *β*_1_-AABs stimulation, dropped to a minimum at 36 h and recovered to normal at 48 h compared with the control group (Figures 4a and c). Beclin-1 mRNA expression significantly decreased 12 h after *β*_1_-AABs stimulation and it was minimal at 36 h and gradually recovered compared with the control group (Figure 4f). Both LC3 protein (see Figures 4a and b) and LC3 mRNA (see Figure 4e) were decreased 12 h after *β*_1_-AABs stimulation, and were minimal at 36 h, returning to normal at 48 h compared with the control group. Immunostaining revealed that green punctate fluorescent signals representing LC3 significantly decreased 36 h after *β*_1_-AABs stimulation, but recovered at 48 h (Figures 4g and h). Thus, *β*_1_-AABs could decrease myocardial autophagy.

In addition, p62 was used to reflect autophagic flux. Figures 4a and d show that p62 protein increased significantly 12 h after *β*_1_-AABs stimulation, peaked at 36 h, and then recovered at 48 h compared with controls. Meanwhile, chloroquine (CQ) was used to block autophagosome–lysosome fusion. Chloroquine rescued LC3 and p62 for 36 h after *β*_1_-AABs stimulation (Figures 4i–k), indicating that autophagic flux decreases in the presence of *β*_1_-AABs.

Cell viability decreased the most 36 h after *β*_1_-AABs stimulation in H9c2 myocardial cells. Therefore, 36 h was selected as the time point for *β*_1_-AABs treatment. Recombinant plasmid pcDNA3.1-Beclin-1 for upregulating autophagy (Supplementary Figures S3a−e) and recombinant plasmid expressing small interfering RNA targeting Beclin-1 (Beclin-1-shRNA) to inhibit autophagy (Supplementary Figures S4a−f) were transfected into H9c2 cells, followed by *β*_1_-AABs stimulation for 36 h. Empty negative control plasmid for protein overexpression and shRNA expression did not influence autophagy (Supplementary Figures S3a−e, S4a−f) and cell viability (Supplementary Figures S3f and S4g). Cell viability increased in cells with upregulated autophagy, but decreased in cells with inhibited autophagy compared with mock cells (Figure 4l). To confirm these data, rapamycin or 3-methyladenine (3-MA) were used to upregulate or suppress autophagy, and cell viability of H9c2 myocardial cells was measured (Figure 4m). Two different ways to increase or inhibit autophagy yielded similar results, suggesting that *β*_1_-AAB-induced decreases in autophagy have a role in cardiomyocyte death.

### Increased autophagy benefitted myocardial cells with early *β*_1_-AABs stimulation

**Figure 5:**
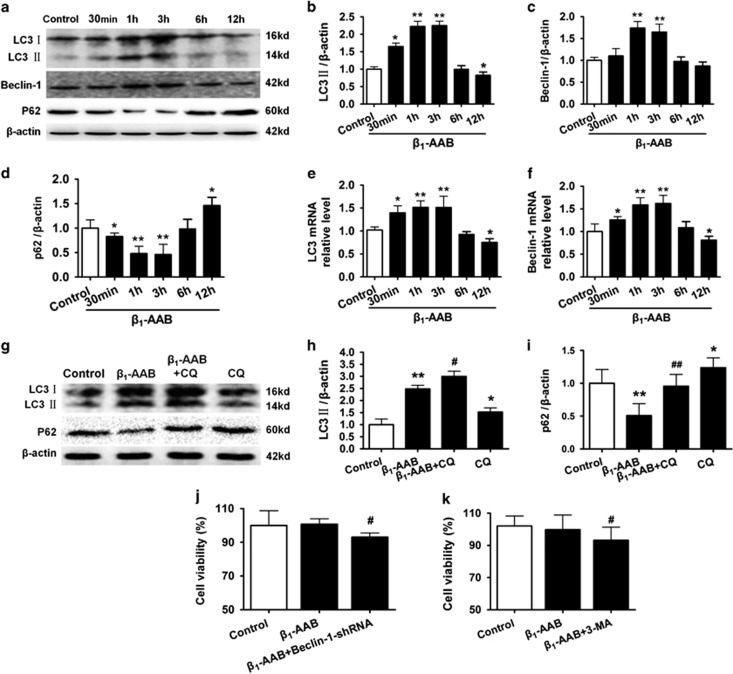
Effect of early *β*_1_-AABs stimulation on autophagy in H9c2 cells and its importance on cardiomyocyte death. (**a**) Representative western blots of autophagy markers LC3, Beclin-1, and p62 at 0, 30 min, 1 h, 3 h, 6 h, and 12 h after *β*_1_-AABs stimulation. (**b**–**d**) Quantification of western blot data from (**a**). Data are expressed as means±S.D. (*n*=6 per group). *P<0.05; **P<0.01. (**e** and **f**) After exposure to *β*_1_-AABs for different time, LC3 and Beclin-1 mRNA were measured by real-time PCR. Data are presented as means±S.D. (*n*=6 per group). *P<0.05; **P<0.01. (**g**) Western blot showed the protein expression of LC3, and p62 after 3 h *β*_1_-AABs stimulation in H9c2 cells pretreated with/or without 20 *μ*M chloroquine (CQ). (**h** and **i**) Quantification of western blot data from (**g**). Data are expressed as means±S.D. (*n*=6 per group). *P<0.05, **P<0.01 *versus* Control; ^#^*P*<0.05, ^##^*P*<0.01 *versus β*_1_-AAB group. (**j** and **k**) Cardiomyocyte death was enhanced by inhibiting autophagy (Beclin1-shRNA or 3-MA) 6 h after *β*_1_-AAB stimulation. Cell viability was determined by CCK-8. (*n*=8, means±S.D.) ^#^*P*<0.05 *versus β*_1_-AAB group

Autophagy is well known as a stress response,^13^ so we observed changes in this stress over time, using earlier *β*_1_-AABs simulation. Cells were collected 0, 30 min, 1, 3, 6, and 12 h after stimulation and LC3 protein, LC3 mRNA, beclin-1 protein, beclin-1 mRNA, and p62 protein were measured. Autophagy increased early as LC3 protein (see Figures 5a and b), LC3 mRNA (see Figure 5e), beclin-1 protein (see Figures 5a and c), and beclin-1 mRNA (see Figure 5f) increased 30 min after *β*_1_-AABs treatment and remained high for 1 and 3 h and recovered to normal at 6 h compared with controls. At 12 h, expression decreased. Meanwhile, p62 protein was declined at 30 min, remained low at 1 and 3 h, returned to normal at 6 h, and then increased at 12 h (Figures 5a and d). These data are consistent with results mentioned above, in which autophagy decreased to the minimum 36 h after *β*_1_-AABs stimulation. Therefore, the presence of *β*_1_-AABs first induced increased autophagic flux in myocardial cells and this may deplete autophagic genes or proteins and cause insufficient autophagy.

To better interpret changes in LC3, chloroquine was used to block the fusion of the autophagosome with the lysosome, and Figures 5g−i show that pretreatment with chloroquine could upregulate LC3 and p62 significantly for 3 h after *β*_1_-AABs stimulation, indicating that early increases in LC3 offer efficient autophagic flux.

To confirm that *β*_1_-AAB induced early increases in autophagy on cardiomyocyte death, autophagy was inhibited with Beclin-1 using RNA interference technology or 3-MA. In addition, because early increased autophagy recovered to almost normal 6 h after stimulation, 6 h was chosen for observation to eliminate the effect of later decreases in autophagy and diminished cell viability. Data show that cell viability did not change 6 h after *β*_1_-AABs stimulation of myocardial cells, but cardiomyocyte death occurred when autophagy was inhibited (Figures 5j and k).

## Discussion

The objective of this study is to explore the importance of *β*_1_-AAB-induced reduction of myocardial autophagy on cardiac function *in vivo*, and discuss changes in *β*_1_-AAB-induced autophagy over time and its significance on cardiomyocyte death. We observed that changes in *β*_1_-AAB-induced autophagy increased early and then decreased. The early increase was cardioprotective, but the later decrease in autophagy prompted cardiomyocyte death and reduced cardiac function *in vivo*.

Recently, autoantibodies against the second extracellular loop of *β*_1_-adrenoceptor (*β*_1_-AR-ECII) (*β*_1_-AABs) were detected in the sera of patients with idiopathic dilated cardiomyopathy,^2^ Chagas' heart disease,^3^ and heart dysfunction caused by ischemic cardiomyopathy.^14^ In our previous study, the long-term presence of autoantibodies may contribute to decreased cardiac function in a rat model immunized with the peptide corresponding to the second extracellular loop of *β*_1_-adrenoceptor.^5^ In this study, a passively immunized rat model was established to eliminate the influence of antigen peptide itself on the body^15^ and this also approximated human *β*_1_-AABs in the rat models.^16^ During model establishment, IgG purified from actively immunized rats was administered to rats to observe the effect of *β*_1_-AABs on cardiac function and we found that *β*_1_-AABs could decrease cardiac function.

It has been reported that reduced autophagy is a major contributor to heart dysfunction,^17^ and upregulated autophagy can significantly improve impaired cardiac function.^18^ Autophagy is a catabolic process by which cells degrade dysfunctional cytoplasmic components and it is necessary for maintenance of cellular homeostasis.^19^ Impaired autophagy causes mitochondrial dysfunction and accumulation, which is closely associated with many human diseases.^11^ Generally, LC3 and Beclin-1 are used to monitor autophagy. Microtubule-associated protein 1 light chain 3 (MAP1-LC3, LC3) is currently a more reliable biomarker to observe autophagy,^20^ and the expression of soluble I-type LC3 (LC3-I) in the cytoplasm is regular. When autophagy occurs, LC3-I converts to LC3-II through ubiquitin-like modification and LC3-II then binds to and localizes in the autophagosomal membrane. LC3-II is well correlated with the number of formed autophagosomes.^21^ As a component of phosphatidylinositol-3-kinase (PI3K) that is necessary in autophagic pathway,^22^ Beclin-1 has an essential role in the formation of autophagosome precursors and membranes,^23^ and is a common index in the observation of autophagy. In addition to LC3 and Beclin-1, p62 are markers for studying autophagic flux.^24^ p62 binds directly to LC3 and is degraded within the autolysosomes,^25^ so accumulation of p62 indicates inhibition of autophagy.^26^ In a previous study, we confirmed that the long-term existence of *β*_1_-AABs reduced autophagy in myocardial tissues.^9^ Similarly, in this study, we also observed that *β*_1_-AABs could decrease myocardial autophagy in a passively immunized rat model. Increased p62 protein indicated a defect in autophagy induced by *β*_1_-AABs. To observe the effect of decreased autophagy on cardiac function, the mTOR inhibitor rapamycin was used to upregulate autophagy. Data show that decreased cardiac function induced by *β*_1_-AABs was effectively reversed by enhanced autophagy. Thus, decreased myocardial autophagy is a major contributor to heart dysfunction.

Myocardial cells are a basic unit of cardiac systolic and diastolic function and when they are damaged or dead, contractile proteins in myocardial cells are degraded immediately, decreasing contractility. To identify a role for *β*_1_-AABs in myocardial cells, we purified IgG antibody in actively immunized rat serum to obtain *β*_1_-AABs. Next, a relatively stable H9c2 cell line was selected from embryonic rat heart tissues, and this line was used to observe the effects of *β*_1_-AABs on survival and autophagy of myocardial cells under the same conditions and time points. Data show that cardiac cell viability deceased at 12 h after *β*_1_-AABs stimulation and was minimal at 36 h, suggesting that *β*_1_-AABs may directly cause cardiomyocyte death. To confirm these data, LDH was measured in myocardial cells because this is documented to leak from damaged cells.^27^ LDH activity in the cell culture medium significantly increased 6 h after *β*_1_-AABs stimulation, indicating damage. In conclusion, *β*_1_-AABs stimulation directly harmed the myocardial cell membrane, and caused cell death. This conclusion is consistent with previous results made by Jane-wit *et al* in an adult rat model.^6^

Previously, *β*_1_-AABs were confirmed to induce apoptosis in cultured neonatal rat myocardial cells.^7^ Staudt's group^28^ also pointed out that *β*_1_-AABs could cause apoptosis in adult isolated myocardial cells. Thus, we measured *β*_1_-AAB effects on myocardial apoptosis over time and found increased apoptosis 6 h after *β*_1_-AABs stimulation and a return to normal at 24 h. Next, the caspase inhibitor Z-VAD-fmk was used to inhibit apoptosis and we observed that cardiomyocyte death recovered to a certain extent 36 h after *β*_1_-AABs stimulation (Supplementary Figure S5), indicating that *β*_1_-AAB-induced apoptosis is involved in cardiomyocyte death. Because *β*_1_-AAB-induced cardiomyocyte death was not completely recovered via apoptotic inhibition, other mechanisms are at play. Thus, we studied H9c2 cells with *β*_1_-AABs at different time points and observed decreased autophagic flux 12 h after stimulation and this was minimal at 36 h, indicating that *β*_1_-AABs stimulation decreased myocardial autophagic flux. Also, comparing changes of autophagy and myocardial cell viability over time after *β*_1_-AABs stimulation, we noted that myocardial cell viability started to decrease 12 h after stimulation, when autophagy decreased dramatically, indicating that the decline of autophagy induced by *β*_1_-AABs may cause cardiomyocyte death. Thus, we used recombinant plasmid pcDNA3.1-Beclin-1 and recombinant plasmid-expressing small interfering RNA targeting Beclin-1 (Beclin-1-shRNA) to upregulate and inhibit myocardial autophagy. Autophagy is reported to be upregulated by increasing or suppressing Beclin-1 expression.^29^ Thus, H9c2 cells were transfected with recombinant plasmid pcDNA3.1-Beclin-1 to upregulate autophagy and we observed that cell viability was higher after *β*_1_-AABs stimulation in transfected cells compared with cells treated with only *β*_1_-AABs. In addition, Beclin-1 RNA interference plasmid was used to transfect H9c2 cells to inhibit autophagy and cell viability decreased after *β*_1_-AABs stimulation. However, beclin-1 could not only induce the autophagy, but also it could suppress autophagosome–lysosome fusion,^30^ so data are difficult to interpret when beclin-1 manipulation is used to modulate autophagy. Therefore, we measured cell viability of H9c2 myocardial cells pretreated with rapamycin to upregulate autophagy or 3-MA to suppress autophagy and both yielded similar results. Therefore, decreased autophagy promotes cardiomyocyte death and improvements in autophagy benefit cardiac function.

Autophagy is commonly regarded as a stress response.^13^ H9c2 myocardial cells were treated with *β*_1_-AABs at earlier time points (0, 30 min, 1, 3, and 6 h) and autophagy was found to first increase and then decrease. However, increased LC3 can be associated with either increased autophagic initiation or reduced degradation in the lysosome. To better distinguish between these two scenarios, chloroquine was applied and data show that the early increase in LC3 means efficient autophagic flux. So the increased autophagy after short-term *β*_1_-AABs stimulation is a stress response, and later depletion of autophagic genes and proteins decreases autophagy. Additional investigations are needed to learn whether *β*_1_-AABs directly induced these changes in myocardial cells. To verify the effect of early increased autophagy after *β*_1_-AABs stimulation on cardiomyocyte death, H9c2 cells were transfected with Beclin-1-shRNA or 3-MA to inhibit autophagy and we found that myocardial survival was significantly reduced after early *β*_1_-AABs stimulation due to inhibition of autophagy. In addition, comparing changes in apoptosis and autophagy over time, autophagy increased before apoptosis. This early increase of autophagy was cardioprotective but this effect gradually disappeared as autophagy decreased and cells died.

In conclusion, autophagy is a stress response before apoptosis in *β*_1_-AAB-induced cardiomyocyte death, and decreased autophagy becomes a subsequent reason for cardiac dysfunction caused by *β*_1_-AAB-induced cardiomyocyte death. Thus, autophagic regulation is more important than apoptosis for patients with *β*_1_-AAB-positive heart dysfunction.

Our study is limited because we only observed that a lack of autophagy caused by *β*_1_-AABs decreased cardiomyocyte death. However, the role of apoptotic changes with autophagic upregulation or inhibition in cardiomyocyte death induced by *β*_1_-AABs requires more study. In addition, validating whether *β*_1_-AAB-induced cardiomyocyte death could be completely reversed by Z-VAD-fmk plus rapamycin is unknown. Still, we conclude that decreased autophagy is a major factor for cardiomyocyte death induced by *β*_1_-AABs. Our preliminary observations may open new insights into the pathogenesis and prevention of *β*_1_-AAB-positive heart dysfunction.

## Materials and Methods

### Animals

Male healthy Wistar rats (8 weeks of age, 140–160 g) obtained from the Animal Center of Shanxi Medical University were used in this study. The experimental procedures were approved by the Institutional Committee on Animal Care of Shanxi Medical University and performed in accordance with the Guide for the Care and Use of Laboratory Animals according to the regulation in the People's Republic of China.

### Extraction of *β*_1_-AABs

First, a *β*_1_-AAB-positive animal model was established by actively immunizing rats with the second extracellular loop antigen peptide of *β*_1_-adrenergic receptor (*β*_1_-AR-ECII), as described in the previous studies.^9^ Then, animal sera from the *β*_1_-AAB-positive group (actively immunized) and the control group were collected and extracted using MAbTrap Kit (GE Healthcare, 17-1128-01, Uppsala, Sweden) for affinity and purification of IgG.

### Passive immunization and rapamycin treatment

Animals were randomized into passive immunization (*β*_1_-AAB) and control (negative IgG) groups. In the *β*_1_-AAB group, *β*_1_-AAB-positive IgG extracted as depicted above was administered to animals via the caudal vein (2 *μ*g/g). This operation was carried out every 10 days and lasted for 80 days. Before each immunization, animal blood samples were collected via tail vein and then the sera were prepared to determine *β*_1_-AABs. In control group, *β*_1_-AAB-negative IgG was administrated by the exact same immunization and determination procedure as *β*_1_-AAB group. A subset of animals were treated with rapamycin (RAPA, Sigma, R0395, St. Louis, MO, USA) in *β*_1_-AAB group (*β*_1_-AAB+RAPA group). RAPA stock solution was prepared by dissolving rapamycin in DMSO (25 mg/ml) and stored until dilution with PBS for intraperitoneal injection. Because *β*_1_-AAB-induced decreases in myocardial autophagy occur on day 20 after passive immunization, RAPA administration started 3 days before this decrease (day 18), beginning at 0.5 mg/kg/day in the first 3 days and then adjusted to 0.25 mg/kg/day^31^ until the end of this study.

### ELISA

ELISA was performed using synthesized peptides corresponding to the sequence of the second extracellular loops of human *β*_1_-adrenoceptors as described previously.^32^ First, 50 *μ*l of the peptide (50 *μ*g/ml) in 100 mM Na_2_CO_3_ solution (pH 11.0) was coated on a microtiter plate (NUNC, Roskilde, Denmark) overnight at 4 °C. Next, the wells were saturated with PMT (PBS containing 3% skimmed milk (W/V), 0.1% Tween 20 (V/V) and 0.01% thimerosal (W/V)) for 1 h at 37 °C. After washing the wells three times with PBS-T, 5 *μ*l of sera was added to 95 *μ*l of PMT solution and incubated for 1 h at 37 °C. After additional washing in PBS-T solution three more times, an affinity-purified biotinylated sheep anti-rat IgG (H+L) antibody (1 : 2000 dilution, Beijing Zhongshan Golden Bridge Biotechnology, ZB-2040, Beijing, China) was added and reacted for 1 h at 37 °C. The plates were washed with PBS-T for three times. The bound biotinylated antibody was detected by incubating the plates with horseradish peroxidase streptavidin (1 : 3000 dilution, Vector, SA-5004, Burlingame, CA, USA) for 1 h. The wells were washed with PBS-T for three times. The substrate (2.5 mM H_2_O_2_, 2 mM 2, 2'-azinodi (ethylbenzthiazoline) sulfuric salt (ABTS, Bio Basic Inc., AD0002, Markham, ON, Canada)) was added. Optical density was measured after 30 min at 405 nm by a microplate reader (Molecular Devices Corp., Sunnyvale, CA, USA). Positive reactions of sera against peptides were verified as described by Liu *et al.*^32^

### *In vivo* measurements of cardiac function

With reference to our previous study,^4^ after anesthesia, a cannula was inserted into the left ventricle via the right carotid artery. BL-410 biological signal recording and analysis system was used to record and analyze the following: the left ventricular systolic pressure (LVSP), left ventricular end diastolic pressure (LVEDP), and maximal positive and negative values of the instantaneous first derivative of left ventricular pressure (+dP/dt_max_ and −dP/dt_max_).

### Cell culture and transfection

Rat myocardial cell-derived cell line H9c2 was purchased from Cell Bank of China Science Academy (Shanghai, China). Cells were cultured in Dulbecco's modified Eagle's medium (DMEM) (Hyclone, SH30022.01B) containing 10% fetal bovine serum (FBS) (Sijiqing, W0001, Hangzhou, China), 100 U/ml penicillin and 100 *μ*g/ml streptomycin (Solarbio, P1400-100, Beijing, China), and incubated in the atmosphere with 5% CO_2_ at 37 °C. Cells were transfected with pcDNA3.1-Beclin-1 (Beclin-1 gene over-expressed, RefSeq Number: NM_053739.2) or Beclin-1-shRNA transfectant (Beclin-1 gene partially-silenced, RefSeq Number: NM_053739.2) using Lipofectamine 2000 (Invitrogen, 11668-027, Grand Island, NY, USA) according to the protocol supplied by the manufacturer. Then, 48–72 h after transfection, mock cells and transfected cells were treated with *β*_1_-AABs (1 *μ*mol/l) or negative IgGs (1 *μ*mol/l) for a specified time. The empty plasmid (control-pcDNA3.1 and control-shRNA) was used as a negative control for protein overexpression and shRNA expression.

### Reagent treatment of H9c2 cells

H9c2 cells were treated with *β*_1_-AABs (1 *μ*mol/l) or negative IgGs (1 *μ*mol/l) for different points or pretreated with 10 ng/ml RAPA or 5 mM 3-MA (Sigma, M9281) or 20 *μ*M chloroquine (Sigma-Aldrich, C6628, St. Louis, MO, USA) for 30 min and then treated with *β*_1_-AABs (1 *μ*mol/l) at different times in the continued presence of RAPA, 3-MA, or chloroquine. The vehicle for RAPA was DMSO (0.1% total volume). 3-MA and chloroquine were dissolved in PBS.

### Measurement of LDH activity

At different time points, a bulk of H9c2 cells were selected and treated with *β*_1_-AABs (*β*_1_-AAB group) or negative IgGs (Control group), and then 0.5 ml of culture supernatant was collected. LDH activity, which reflects the extent of cellular damage, was measured with a colorimetric assay kit (Nanjing Jiancheng Biochemical Reagent Co., Nanjing, China) according to the protocol supplied by the manufacturer. Assay sample absorbances were measured at 450 nm.

### Cell viability assay

Cell viability was measured with a cell counting kit-8 (CCK-8). H9c2 cells were inoculated in 96-well plates (1 × 10^4^ cells) and cells were treated with *β*_1_-AABs at different time points. Then, 10 *μ*l CCK-8 (Dojindo Molecular Technologies, CK04, Kumamoto, Japan) was added to the plates and cells were incubated for 4 h at 37 °C. Sample absorbance was measured at 450 nm with a microplate reader by the following equation: viability %=[(AS−AB)/(AC−AB)] × 100%, where AS is the absorbance of the samples with *β*_1_-AABs, AC is the absorbance of the DMEM media, and AB is the absorbance of the control.

### LC3, Beclin-1, and p62 protein measurement by western blot

Western blot was used to quantify LC3 and Beclin-1 expression in tissues and cells. After specific treatments, cardiac tissue (70 mg) was lysed in RIPA buffer (Beyotime Biotech, P0013B, Haimen, China) or total proteins were isolated from cells using cell lysis buffer (Cell Signaling, #9803, Beverly, MA, USA). Then, supernatant fluids were collected after centrifugation and protein was quantified with BCA Protein Assay Kit (Thermo Scientific, 23225, Rockford, IL, USA). First, 40 mg of sample was loaded and separated by SDS-PAGE and then transferred onto a PVDF membrane (Millipore, IPVH00010, Billerica, MA, USA) followed by addition of primary antibodies against anti-Beclin-1 monoclonal (1 : 1000; Santa Cruz, sc-48381, Dallas, TX, USA), anti-LC3B monoclonal (1 : 1000; Cell Signaling Tech, 2775, Beverly, MA, USA), anti-SQSTM1/p62 polyclonal (1 : 1000; Cell Signaling Tech, 5114) and anti-*β*-actin monoclonal (1 : 1000; Sigma-Aldrich, A1978) at 4 °C overnight. Next, the membrane was blotted with the corresponding secondary antibody for 1 h (1 : 3000; Beijing Zhongshan Golden Bridge Biotechnology, ZB-2305, ZB-2301). The blot was developed using a SuperEnhanced chemiluminescent detection kit, and then the membrane was placed in a Kodak Image Station 400 (Eastman Kodak Co., Rochester, NY, USA) for exposure and images of membrane signal bands were obtained using Kodak ID Software (Eastman Kodak Co.).

### LC3 and Beclin-1 mRNA measurement with real-time PCR

Real-time PCR was used to measure LC3 and Beclin-1 mRNA in H9c2 cells. Approximately 3 *μ*g of total RNA was extracted from cells and reverse transcribed to cDNA. The thermal profile of SYBR Green PCR included 30 s heat activation of the enzyme at 95 °C followed by 40 cycles of denaturation at 95 °C for 5 s and annealing/extension at 60 °C for 20 s. The primer sequences were as follows: LC3, sense: 5′-CATGCCGTCCGAGAAGACCT-3′ and antisense: 5′-GATGAGCCGGACATCTTCCACT-3′ (GenBank accession number, NM022867.2); Beclin-1, sense: 5′-TTGGCCAATAAGATGGGTCTGAA-3′ and antisense: 5′-TGTCAGGGACTCCAGATACGAGTG-3′ (GenBank accession number, NM001034117.1). Expression was standardized to GAPDH and data, expressed as a fold-difference in the number of LC3 or Beclin-1 copies relative to the number of GAPDH copies, were quantified by the relative quantitative 2^−ΔΔCt^ method. ΔΔCt=ΔCt (target gene)−ΔCt (GAPDH); ΔCt (target gene)=Ct (experimental−target)−Ct (control−target); and ΔCt (GAPDH)=Ct (experimental−GAPDH)−Ct (control−GAPDH).

### Cell immunostaining

The *in situ* expression of LC3 protein in H9c2 cells was observed with immunostaining. H9c2 cells were extracted from the medium and washed twice with precooled PBS. After fixation in 4% paraformaldehyde for 15 min, cells were washed with 0.2% Triton in PBS three times for 3 min each time and then blocked with immunostaining blocking buffer (Beyotime Biotech, P0102) at room temperature for 1 h. Primary LC3B antibody was added to the section (1:400, Cell Signaling Tech, 2775) at 4 °C overnight and subsequently reacted with FITC-labeled secondary fluorescence antibody of the corresponding species (1 : 50, Beijing Zhongshan Golden Bridge Biotechnology, ZF-0311). DAPI (Beyotime Biotech, C1005) was used to stain nuclei for 3 min. Sections were mounted and observed under laser confocal microscopy (OLYMPUS, FV1000, Tokyo, Japan). Mean LC3 puncta per cell quantified from 20 different fields was measured by manual counting and DAPI-stained nuclei were counted as cells in the same fields. Then, LC3 puncta per cell were calculated by dividing total dot numbers by quantified nuclei per microscopic field.

### Statistical analysis

Data are expressed as means±standard deviation (S.D.). Statistical analysis was performed with SPSS software (version 15.0, SPSS Inc., Chicago, IL, USA). A Student's *t*-test was used to compare the means of two independent samples and one-way ANOVA was applied after a Bonferroni *post hoc* test for more than two samples. Data were considered as statistically significant when *P*<0.05.

## Abbreviations

*β*_1_-AABs: anti-*β*_1_-adrenergic receptor autoantibodies
RNAi: RNA interference
LVSP: left ventricular systolic pressure
+dP/dt_max_: maximal positive values of the instantaneous first derivative of left ventricular pressure
−dP/dt_max_: maximal negative values of the instantaneous first derivative of left ventricular pressure
LVEDP: left ventricular end diastolic pressure
RAPA: rapamycin
CQ: chloroquine
3-MA: 3-methyladenine
*β*_1_-AR-ECII: the second extracellular loop of *β*_1_-adrenoceptor
MAP1-LC3: LC3, microtubule-associated protein 1 light chain 3
PI3K: phosphatidylinositol-3-kinase
DMEM: Dulbecco's modified Eagle's medium
FBS: fetal bovine serum
CCK-8: cell counting kit-8

## References

[bib1] Yamada T Matsumori A Wang WZ Ohashi N Shiota K Sasayama S Apoptosis in congestive heart failure induced by viral myocarditis in mice Heart Vessels 1999 14 29 37 10543311 10.1007/BF02481740

[bib2] Magnusson Y Wallukat G Waagstein F Hjalmarson A Hoebeke J Autoimmunity in idiopathic dilated cardiomyopathy. Characterization of antibodies against the beta 1-adrenoceptor with positive chronotropic effect Circulation 1994 89 2760 2767 8205690 10.1161/01.cir.89.6.2760

[bib3] Rosenbaum MB Chiale PA Schejtman D Levin M Elizari MV Antibodies to beta-adrenergic receptors disclosing agonist-like properties in idiopathic dilated cardiomyopathy and Chagas' heart disease J Cardiovasc Electrophysiol 1994 5 367 375 8019712 10.1111/j.1540-8167.1994.tb01174.x

[bib4] Liu HR Zhao RR Jiao XY Wang YY Fu M Relationship of myocardial remodeling to the genesis of serum autoantibodies to cardiac beta(1)-adrenoceptors and muscarinic type 2 acetylcholine receptors in rats J Am Coll Cardiol 2002 39 1866 1873 12039504 10.1016/s0735-1097(02)01865-x

[bib5] Zuo L Bao H Tian J Wang X Zhang S He Z Long-term active immunization with a synthetic peptide corresponding to the second extracellular loop of beta1-adrenoceptor induces both morphological and functional cardiomyopathic changes in rats Int J Cardiol 2011 149 89 94 20096470 10.1016/j.ijcard.2009.12.023

[bib6] Jane-wit D Altuntas CZ Johnson JM Yong S Wickley PJ Clark P Beta 1-adrenergic receptor autoantibodies mediate dilated cardiomyopathy by agonistically inducing cardiomyocyte apoptosis Circulation 2007 116 399 410 17620508 10.1161/CIRCULATIONAHA.106.683193

[bib7] Gao Y Liu HR Zhao RR Zhi JM Autoantibody against cardiac beta1-adrenoceptor induces apoptosis in cultured neonatal rat cardiomyocytes Acta Biochim Biophys Sin 2006 38 443 449 16820859 10.1111/j.1745-7270.2006.00185.x

[bib8] Mao W Fukuoka S Iwai C Liu J Sharma VK Sheu SS Cardiomyocyte apoptosis in autoimmune cardiomyopathy: mediated via endoplasmic reticulum stress and exaggerated by norepinephrine Am J Physiol Heart Circ Physiol 2007 293 H1636 H1645 17545481 10.1152/ajpheart.01377.2006

[bib9] Wang L Lu K Hao H Li X Wang J Wang K Decreased autophagy in rat heart induced by anti-beta1-adrenergic receptor autoantibodies contributes to the decline in mitochondrial membrane potential PLoS ONE 2013 8 e81296 24278413 10.1371/journal.pone.0081296 PMC3835737

[bib10] Komatsu M Ueno T Waguri S Uchiyama Y Kominami E Tanaka K Constitutive autophagy: vital role in clearance of unfavorable proteins in neurons Cell Death Differ 2007 14 887 894 17332773 10.1038/sj.cdd.4402120

[bib11] Wrighton KH Autophagy: shaping the fate of mitochondria Nat Rev Mol Cell Biol 2011 12 344 345 21527952 10.1038/nrm3116

[bib12] Meijer AJ Codogno P Regulation and role of autophagy in mammalian cells Int J Biochem Cell Biol 2004 36 2445 2462 15325584 10.1016/j.biocel.2004.02.002

[bib13] Kim J Kim YC Fang C Russell RC Kim JH Fan W Differential regulation of distinct Vps34 complexes by AMPK in nutrient stress and autophagy Cell 2013 152 290 303 23332761 10.1016/j.cell.2012.12.016 PMC3587159

[bib14] Zhang L Hu D Shi X Li J Zeng W Xu L Autoantibodies against the myocardium beta 1-adrenergic and M2-muscarinic receptors in patients with heart failure Zhonghua Nei Ke Za Zhi 2001 40 445 447 11798611

[bib15] Jahns R Boivin V Hein L Triebel S Angermann CE Ertl G Direct evidence for a beta 1-adrenergic receptor-directed autoimmune attack as a cause of idiopathic dilated cardiomyopathy J Clin Invest 2004 113 1419 1429 15146239 10.1172/JCI20149 PMC406525

[bib16] Du Y Yan L Du H Wang L Ding F Quan L beta1 -adrenergic receptor autoantibodies from heart failure patients enhanced TNF-alpha secretion in RAW264.7 macrophages in a largely PKA-dependent fashion J Cell Biochem 2012 113 3218 3228 22628174 10.1002/jcb.24198

[bib17] Sun A Cheng Y Zhang Y Zhang Q Wang S Tian S Aldehyde dehydrogenase 2 ameliorates doxorubicin-induced myocardial dysfunction through detoxification of 4-HNE and suppression of autophagy J Mol Cell Cardiol 2014 71 92 104 24434637 10.1016/j.yjmcc.2014.01.002

[bib18] Bhuiyan MS Pattison JS Osinska H James J Gulick J McLendon PM Enhanced autophagy ameliorates cardiac proteinopathy J Clin Invest 2013 123 5284 5297 24177425 10.1172/JCI70877 PMC3859422

[bib19] Klionsky DJ Emr SD Autophagy as a regulated pathway of cellular degradation Science 2000 290 1717 1721 11099404 10.1126/science.290.5497.1717 PMC2732363

[bib20] Mizushima N Methods for monitoring autophagy Int J Biochem Cell Biol 2004 36 2491 2502 15325587 10.1016/j.biocel.2004.02.005

[bib21] Shintani T Klionsky DJ Autophagy in health and disease: a double-edged sword Science 2004 306 990 995 15528435 10.1126/science.1099993 PMC1705980

[bib22] Liang XH Jackson S Seaman M Brown K Kempkes B Hibshoosh H Induction of autophagy and inhibition of tumorigenesis by beclin 1 Nature 1999 402 672 676 10604474 10.1038/45257

[bib23] Takahashi Y Coppola D Matsushita N Cualing HD Sun M Sato Y Bif-1 interacts with Beclin 1 through UVRAG and regulates autophagy and tumorigenesis Nat Cell Biol 2007 9 1142 1151 17891140 10.1038/ncb1634 PMC2254521

[bib24] Klionsky DJ Abdalla FC Abeliovich H Abraham RT Acevedo-Arozena A Adeli K Guidelines for the use and interpretation of assays for monitoring autophagy Autophagy 2012 8 445 544 22966490 10.4161/auto.19496 PMC3404883

[bib25] Bjørkøy G Lamark T Brech A Outzen H Perander M Øvervatn A p62/SQSTM1 forms protein aggregates degraded by autophagy and has a protective effect on huntingtin-induced cell death J Cell Biol 2005 171 603 614 16286508 10.1083/jcb.200507002 PMC2171557

[bib26] Komatsu M Waguri S Koike M Sou YS Ueno T Hara T Homeostatic levels of p62 control cytoplasmic inclusion body formation in autophagy-deficient mice Cell 2007 131 1149 1163 18083104 10.1016/j.cell.2007.10.035

[bib27] Sun HY Wang NP Kerendi F Halkos M Kin H Guyton RA Hypoxic postconditioning reduces cardiomyocyte loss by inhibiting ROS generation and intracellular Ca2+ overload Am J Physiol Heart Circ Physiol 2005 288 H1900 H1908 15563525 10.1152/ajpheart.01244.2003

[bib28] Staudt Y Mobini R Fu M Felix SB Kuhn JP Staudt A Beta1-adrenoceptor antibodies induce apoptosis in adult isolated cardiomyocytes Eur J Pharmacol 2003 466 1 6 12679135 10.1016/s0014-2999(03)01431-6

[bib29] Zhu H Tannous P Johnstone JL Kong Y Shelton JM Richardson JA Cardiac autophagy is a maladaptive response to hemodynamic stress J Clin Invest 2007 117 1782 1793 10.1172/JCI27523 PMC1890995 17607355

[bib30] Gottlieb RA Andres AM Sin J Taylor DP Untangling autophagy measurements: all fluxed up Circ Res 2015 116 504 514 25634973 10.1161/CIRCRESAHA.116.303787 PMC4313387

[bib31] Gallo R Padurean A Jayaraman T Marx S Roque M Adelman S Inhibition of intimal thickening after balloon angioplasty in porcine coronary arteries by targeting regulators of the cell cycle Circulation 1999 99 2164 2170 10.1161/01.cir.99.16.2164 10217658

[bib32] Liu HR Zhao RR Zhi JM Wu BW Fu ML Screening of serum autoantibodies to cardiac beta1-adrenoceptors and M2-muscarinic acetylcholine receptors in 408 healthy subjects of varying ages Autoimmunity 1999 29 43 51 10.3109/08916939908995971 10052684

