# Supplementary material for: Decreased autophagy: a major factor for cardiomyocyte death induced by β1-adrenoceptor autoantibodies
Source: Cell Death Dis. 2015 Aug 27;6(8):e1862–. doi: 10.1038/cddis.2015.237 (PMC4558518; doi:10.1038/cddis.2015.237)
Supplement: Supplementary Figure Legends [file cddis2015237x1.doc]

**Supplementary Figure Legends**

**Figure S1** Measurement of cardiac function in passively immunized rats at different time points. (a) Serum β_1_-AABs at different time points after passively immunized with purified β_1_-AABs by tail vein. Antibody titer was defined by OD values. Data are expressed as means ± S.D. (n = 16 per group). ^**^P < 0.01. (b-e) Rats had less cardiac function 80 days after passive immunization with β_1_-AABs. Cardiac function was measured with LVSP, LVEDP, +dp/dt_max_ and −dp/dt_max_. Data are expressed as means ± S.D. (n = 6 per group). ^*^P < 0.05; ^**^P < 0.01.

**Figure S2** Effect of rapamycin (RAPA) on myocardial autophagy in passively immunized rats. (a) Representative Western blots of autophagic markers LC3 and Beclin-1. (b, c) Quantification of Western blot data from a. Data are expressed as means ± S.D. (n = 6 per group). ^**^P < 0.01 vs. Control; ^#^P < 0.05 vs. β_1_-AAB group.

**Figure S3** Effect of recombinant plasmid pcDNA3.1-Beclin-1 overexpressing Beclin-1 and the empty plasmid control-pcDNA3.1 on autophagy and cell viability in transfected H9c2 cells. (a, b) Real-time PCR analysis confirmed LC3 and Beclin-1 mRNA expression in H9c2 cells. Data are expressed as means ± S.D. (n = 6 per group). ^**^P < 0.01. (c) Representative Western blots of LC3 and Beclin-1 after transfecting H9c2 cells with pcDNA3.1-Beclin-1 and control-pcDNA3.1. (d, e) Quantification of western blot data from a. Data were represented means ± S.D. (n = 6 per group). ^**^P < 0.01. (f) The empty plasmid control-pcDNA3.1 have no effect on cell viability. Data were represented means ± S.D. (n = 8 per group).

**Figure S4** Effect of recombinant plasmid Beclin-1-shRNA using RNA interference technology and the empty plasmid control-shRNA on autophagy and cell viability in transfected H9c2 cells. (a, b) Real-time PCR analysis showed the LC3 and Beclin-1 mRNA expression in H9c2 cells. Data are expressed as means ± S.D. (n = 6 per group). ^**^P < 0.01. (c) Representative Western blots of LC3 and Beclin-1 after transfecting H9c2 cells with Beclin-1-shRNA and control-shRNA. (d, e) Quantification of Western blot data from a. Data represent means ± S.D. (n = 6 per group). ^**^P < 0.01. (f) Representative green immunofluorescence for H9c2 cells transfected with Beclin-1-shRNA. The immunofluorescence signal showed the enhanced green fluorescent protein (EGFP) and reflected successful transfection. When the shRNA vector together with EGFP (U6-shRNA-CMV-EGFP-PGK-puro) was transfected to cells, the green immunofluorescence signal emerged. (g) The empty plasmid control-shRNA have no effect on cell viability. Data were represented means ± S.D. (n = 8 per group).

**Figure S5** β_1_-AAB-induced cardiomyocyte death is partially improved by inhibiting apoptosis. (a) Representative flow cytometric analysis using Annexin V-FITC Apoptosis Detection Kit for detection of apoptosis in myocardial cells after β_1_-AABs stimulation. Cells in the lower left quadrant (Annexin V-FITC−/PI−) are viable, those in the lower right quadrant (Annexin V-FITC+/PI−) are early apoptotic and those in the upper right quadrants (Annexin V-FITC+/PI+) are late apoptotic or necrotic. Therefore, lower right quadrant cells what we concern shows the percentage of apoptotic cells and do not include necrotic cells. (b) Quantification of data from the lower right quadrant of a. Data are expressed as means ± S.D. (n = 6 per group). ^*^P < 0.05;^**^P < 0.01. (c) Caspase-3 Activity Assay was used to measure apoptosis in H9c2 cells stimulated by β_1_-AABs. Data are expressed as means ± S.D. (n = 6 per group). ^*^P < 0.05; ^**^P < 0.01. (d) Caspase inhibitor Z-VAD-fmk significantly inhibited caspase-3 activity in H9c2 cells at 6 h after stimulation by β_1_-AABs. Data are expressed as means ± S.D. (n = 6 per group). ^**^P < 0.01 vs. Control; ^#^P < 0.05 vs. β_1_-AAB group. (e) β_1_-AAB-induced decrease of myocardial cells is partially recovered by inhibiting apoptosis after 36 h β_1_-AABs stimulation. Data are expressed as means ± S.D. (n = 8 per group). ^**^P < 0.01 vs. Control; ^#^P < 0.05 vs. β_1_-AAB group.
