# Supplementary figures and images for: Decreased autophagy: a major factor for cardiomyocyte death induced by β1-adrenoceptor autoantibodies
Source: Cell Death Dis. 2015 Aug 27;6(8):e1862–. doi: 10.1038/cddis.2015.237 (PMC4558518; doi:10.1038/cddis.2015.237)

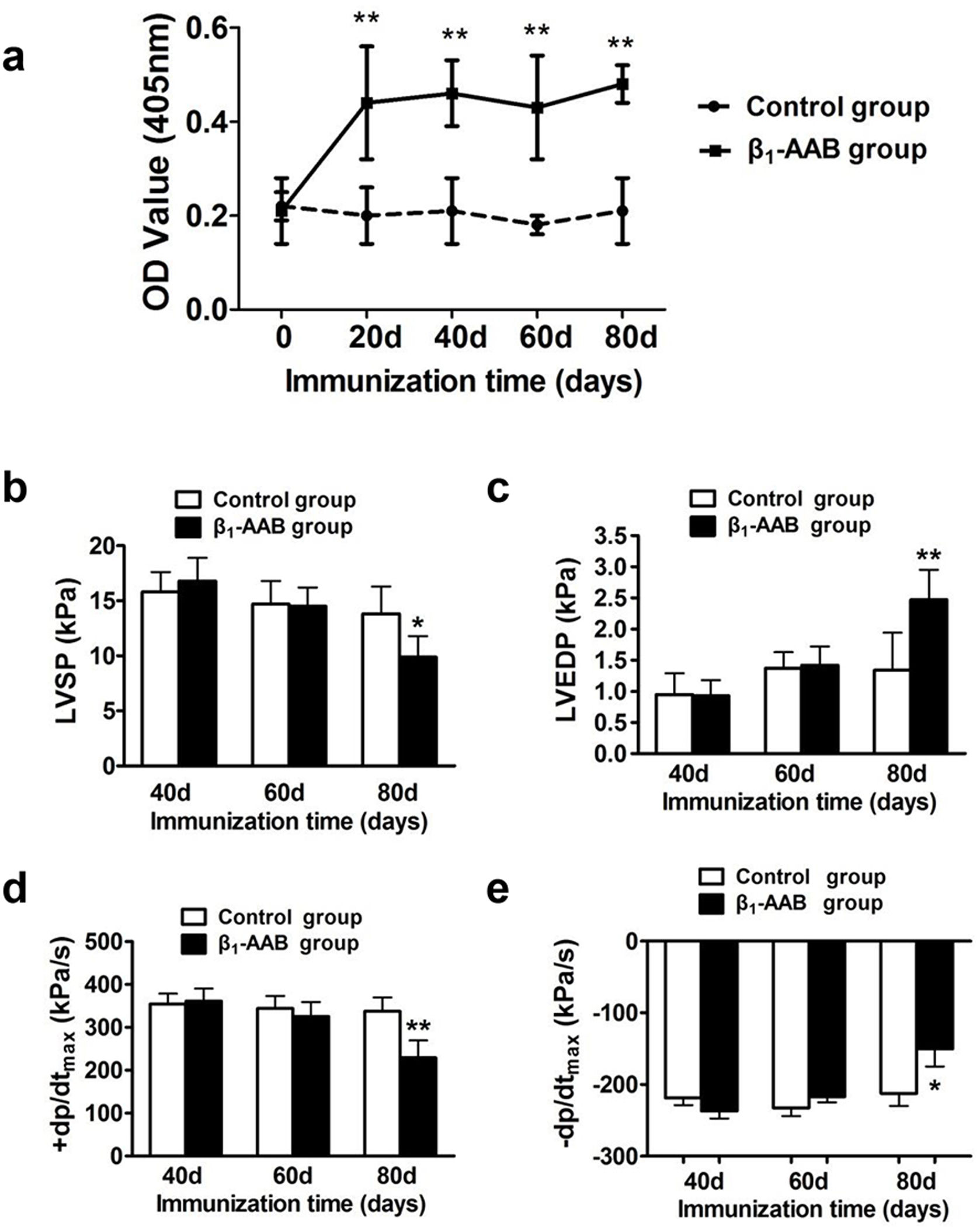

Supplement: Supplementary Figure 1 [file cddis2015237x2.tif]

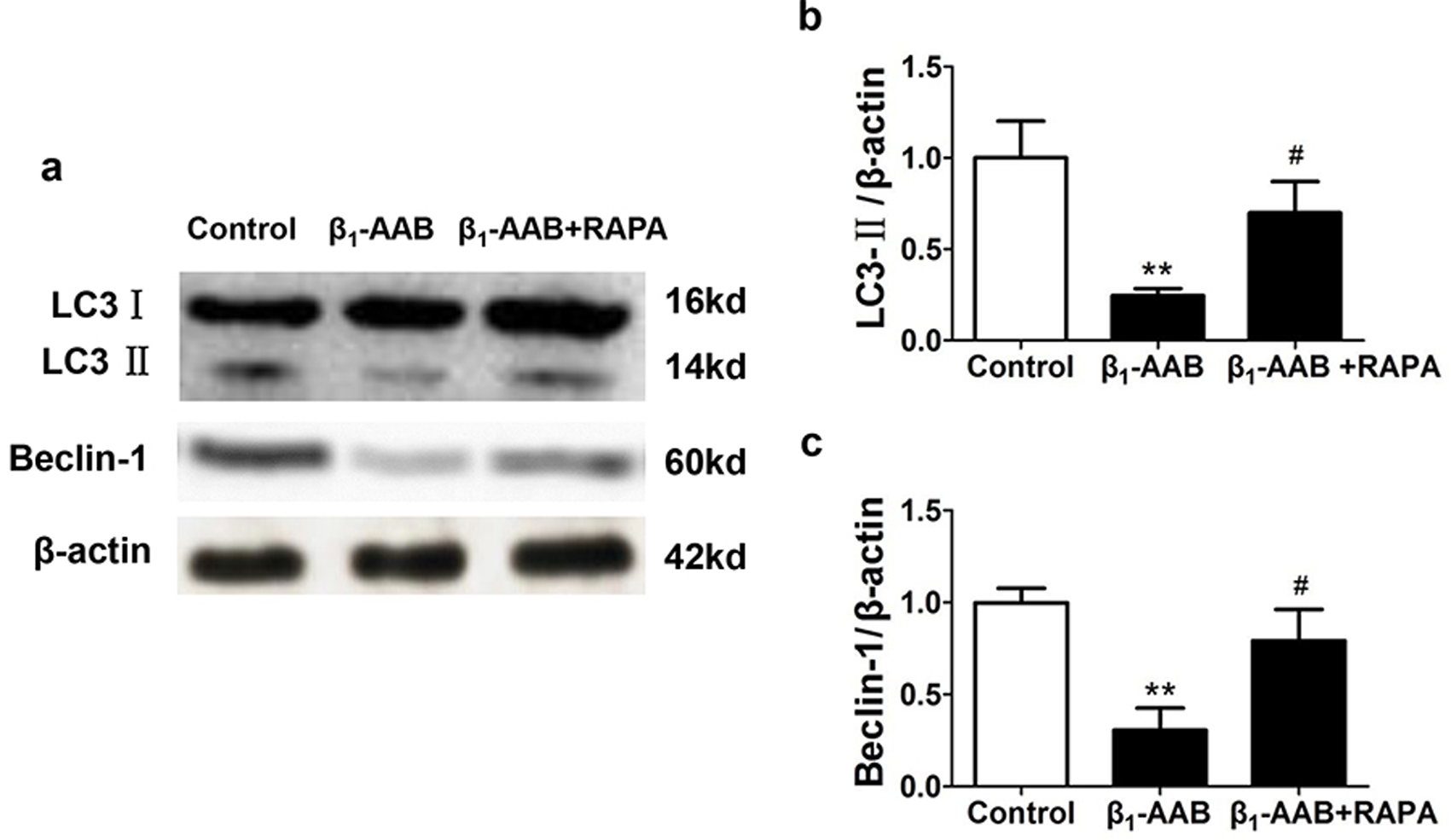

Supplement: Supplementary Figure 2 [file cddis2015237x3.tif]

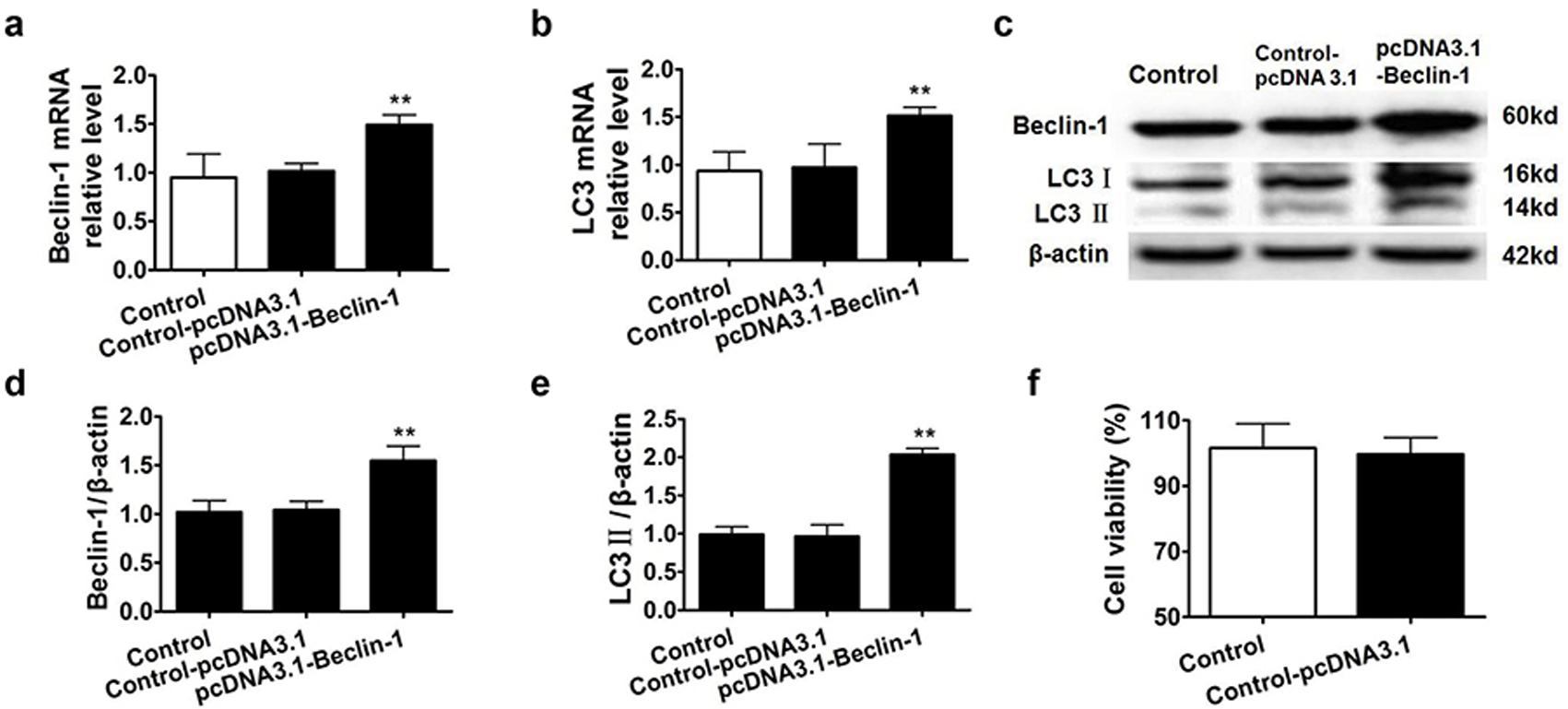

Supplement: Supplementary Figure 3 [file cddis2015237x4.tif]

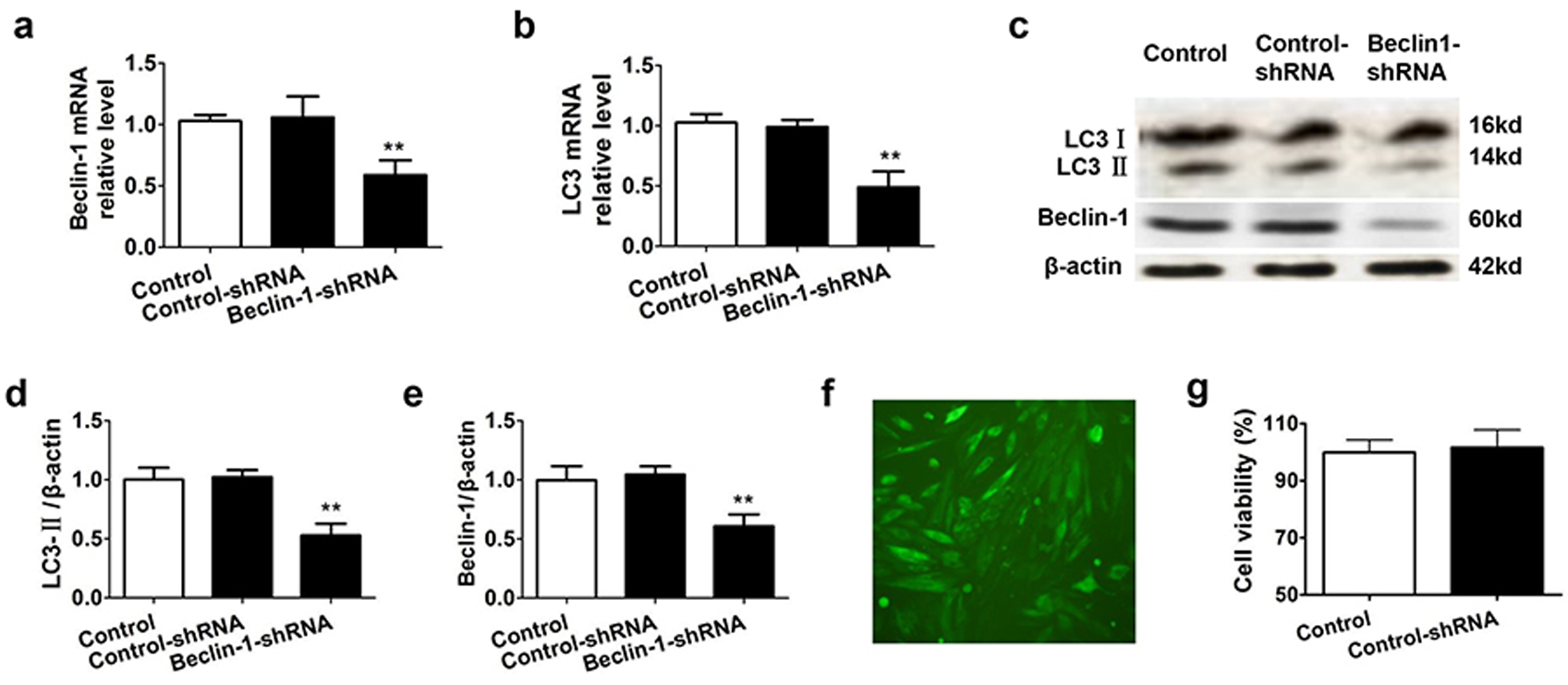

Supplement: Supplementary Figure 4 [file cddis2015237x5.tif]

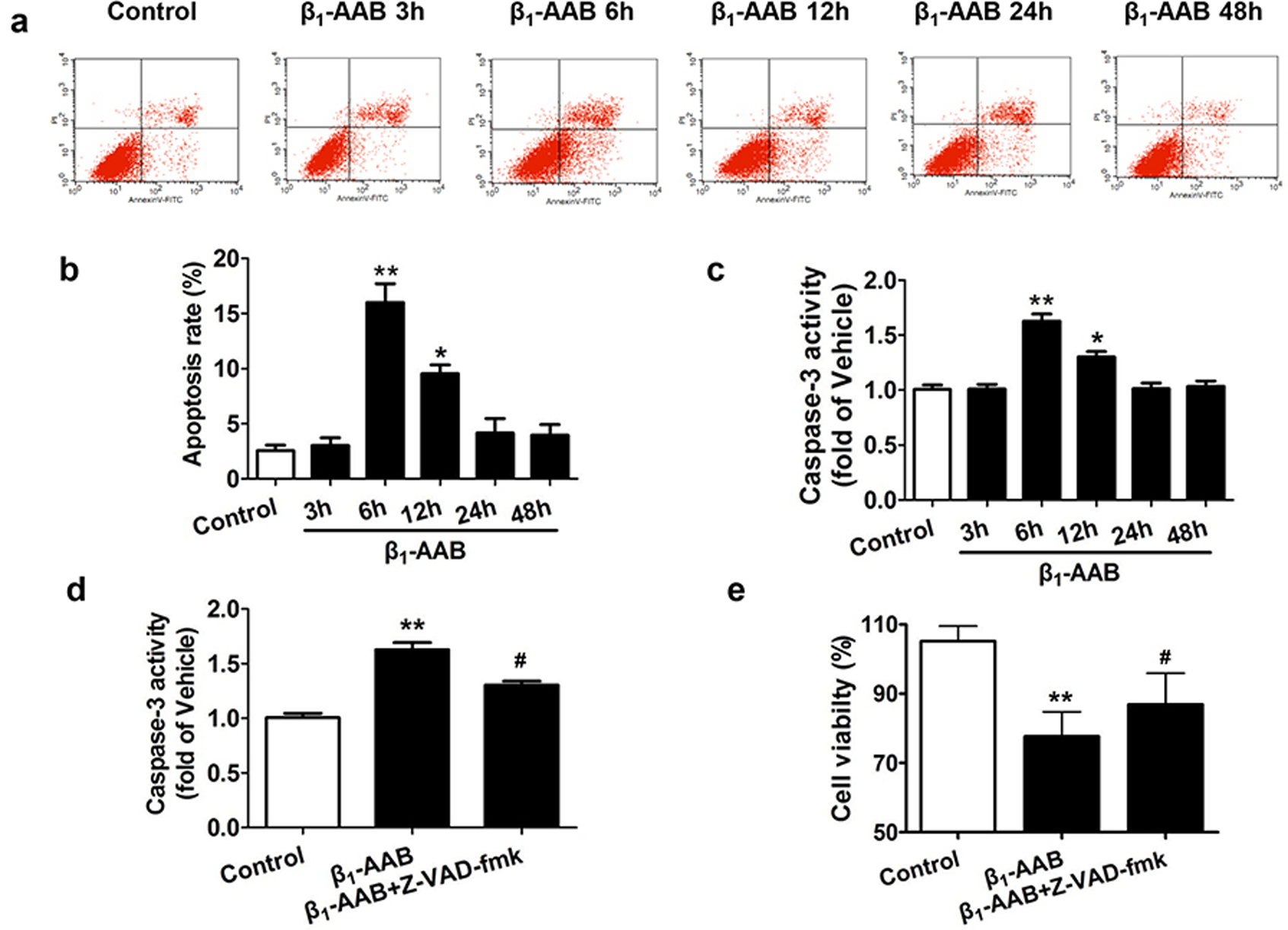

Supplement: Supplementary Figure 5 [file cddis2015237x6.tif]
